# 4-Chloro-*N*′-(5-chloro-2-hydroxy­benzyl­idene)benzohydrazide

**DOI:** 10.1107/S1600536808025816

**Published:** 2008-08-16

**Authors:** De-Suo Yang

**Affiliations:** a Department of Chemistry and Chemical Engineering, Baoji University of Arts and Sciences, Baoji 721007, People’s Republic of China

## Abstract

The mol­ecule of the title compound, C_14_H_10_Cl_2_N_2_O_2_, displays a *trans* configuration with respect to the C=N double bond and has an intramolecular O—H⋯N hydrogen bond. The dihedral angle between the two benzene rings is 1.4 (2)°. In the crystal structure, mol­ecules are linked through inter­molecular N—H⋯O hydrogen bonds, forming chains running along the *a* direction.

## Related literature

For related structures, see Yang (2006*a* ▶,*b* ▶,*c* ▶,*d* ▶,*e* ▶, 2007*a* ▶,*b* ▶,*c* ▶); Yang & Guo (2006 ▶). For related literature, see: Allen *et al.* (1987 ▶); Bernardo *et al.* (1996 ▶); Musie *et al.* (2001 ▶); Paul *et al.* (2002 ▶).
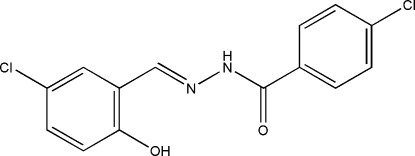


## Experimental

### 

#### Crystal data

C_14_H_10_Cl_2_N_2_O_2_
*M* *_r_* = 309.14
Monoclinic, 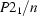

*a* = 5.921 (2) Å
*b* = 31.245 (3) Å
*c* = 7.428 (3) Å
β = 92.182 (6)°
*V* = 1373.2 (7) Å^3^
*Z* = 4
Mo *K*α radiation
μ = 0.47 mm^−1^
*T* = 298 (2) K
0.20 × 0.18 × 0.17 mm

#### Data collection

Bruker SMART CCD diffractometer
Absorption correction: multi-scan (*SADABS*; Sheldrick, 1996 ▶) *T*_min_ = 0.911, *T*_max_ = 0.924
6465 measured reflections
2239 independent reflections
1790 reflections with *I* > 2σ(*I*)
*R*_int_ = 0.022

#### Refinement

*R*[*F* ^2^ > 2σ(*F* ^2^)] = 0.037
*wR*(*F* ^2^) = 0.097
*S* = 1.03
2239 reflections
185 parameters
1 restraint
H atoms treated by a mixture of independent and constrained refinement
Δρ_max_ = 0.24 e Å^−3^
Δρ_min_ = −0.25 e Å^−3^

### 

Data collection: *SMART* (Bruker, 2007 ▶); cell refinement: *SAINT* (Bruker, 2007 ▶); data reduction: *SAINT*; program(s) used to solve structure: *SHELXS97* (Sheldrick, 2008 ▶); program(s) used to refine structure: *SHELXL97* (Sheldrick, 2008 ▶); molecular graphics: *SHELXTL* (Sheldrick, 2008 ▶); software used to prepare material for publication: *SHELXL97*.

## Supplementary Material

Crystal structure: contains datablocks global, I. DOI: 10.1107/S1600536808025816/om2256sup1.cif

Structure factors: contains datablocks I. DOI: 10.1107/S1600536808025816/om2256Isup2.hkl

Additional supplementary materials:  crystallographic information; 3D view; checkCIF report

## Figures and Tables

**Table 1:** Hydrogen-bond geometry (Å, °)

*D*—H⋯*A*	*D*—H	H⋯*A*	*D*⋯*A*	*D*—H⋯*A*
N2—H2⋯O2^i^	0.898 (10)	1.965 (13)	2.826 (2)	160 (2)
O1—H1⋯N1	0.82	1.93	2.647 (2)	145

Symmetry code: (i) 
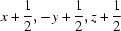
.

**Fig. 1.**
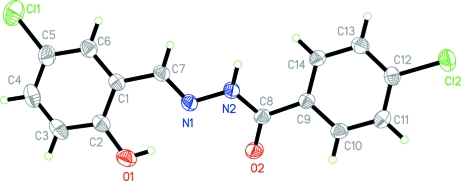
The structure of the title compound showing the atom-numbering scheme. Displacement ellipsoids are drawn at the 30% probability level.

**Fig. 2.**
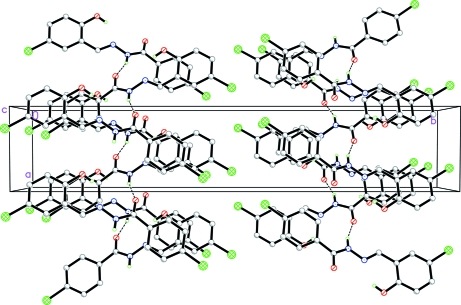
Molecular packing as viewed along the c axis. Hydrogen bonds are shown as dashed lines.

## supplementary crystallographic information

### Comment

Schiff base compounds have been of great interest for a long time. These compounds play an important role in the development of coordination chemistry (Musie *et al.*, 2001; Bernardo *et al.*, 1996; Paul *et al.*, 2002). Recently, we have reported a few Schiff base compounds (Yang, 2006*a*,*b*,*c*,*d*,*e*, 2007*a*,*b*,*c*; Yang & Guo, 2006). As a further investigation of this work, the crystal structure of the title compound is reported here.

The molecule of the title compound, displays a *trans* configuration with respect to the C═N double bond (Fig. 1). The dihedral angle between the two benzene rings is 1.4 (2)°. All the bond lengths are within normal ranges (Allen *et al.*, 1987). The C7═N1 bond length of 1.270 (3) Å conforms to the value for a double bond. The bond length of 1.343 (3) Å between atoms C8 and N2 is intermediate between a N—N single bond and a N═N double bond, because of conjugation effects in the molecule. There is a strong intramolecular hydrogen bond between the hydroxyl hydrogen and N1.

In the crystal structure, molecules are linked through intermolecular N—H···O hydrogen bonds (Table 1), forming chains running along the *a* direction (Fig. 2).

### Experimental

5-Chlorosalicylaldehyde (0.1 mmol, 15.6 mg) and 4-chlorobenzohydrazide (0.1 mmol, 17.0 mg) were dissolved in MeOH (10 ml). The mixture was stirred at room temperature to give a clear colorless solution. Crystals of the title compound were formed by gradual evaporation of the solvent over a period of 13 days at room temperature.

### Refinement

Atom H2 was located in a difference Fourier map and refined isotropically, with N—H distance restrained to 0.90 (1) Å. Other H atoms were placed in idealized positions and constrained to ride on their parent atoms, with O—H distance of 0.82 Å, C—H distances of 0.93 Å, and with *U*_iso_(H) = 1.2*U*_eq_(C) and 1.5*U*_eq_(O).

### Crystal data

**Table:**

C_14_H_10_Cl_2_N_2_O_2_	*F*_000_ = 632
*M**_r_* = 309.14	*D*_x_ = 1.495 Mg m^−^^3^
Monoclinic, *P*2_1_/*n*	Mo *K*α radiation λ = 0.71073 Å
Hall symbol: -P 2yn	Cell parameters from 2553 reflections
*a* = 5.921 (2) Å	θ = 2.5–24.3º
*b* = 31.245 (3) Å	µ = 0.47 mm^−^^1^
*c* = 7.428 (3) Å	*T* = 298 (2) K
β = 92.182 (6)º	Block, colourless
*V* = 1373.2 (7) Å^3^	0.20 × 0.18 × 0.17 mm
*Z* = 4	

### Data collection

**Table:**

Bruker SMART CCD diffractometer	2239 independent reflections
Radiation source: fine-focus sealed tube	1790 reflections with *I* > 2σ(*I*)
Monochromator: graphite	*R*_int_ = 0.022
*T* = 298(2) K	θ_max_ = 24.4º
ω scans	θ_min_ = 2.6º
Absorption correction: multi-scan(SADABS; Sheldrick, 1996)	*h* = −6→6
*T*_min_ = 0.911, *T*_max_ = 0.924	*k* = −36→35
6465 measured reflections	*l* = −8→6

### Refinement

**Table:**

Refinement on *F*^2^	Secondary atom site location: difference Fourier map
Least-squares matrix: full	Hydrogen site location: inferred from neighbouring sites
*R*[*F*^2^ > 2σ(*F*^2^)] = 0.038	H atoms treated by a mixture of independent and constrained refinement
*wR*(*F*^2^) = 0.097	*w* = 1/[σ^2^(*F*_o_^2^) + (0.0381*P*)^2^ + 0.5294*P*] where *P* = (*F*_o_^2^ + 2*F*_c_^2^)/3
*S* = 1.03	(Δ/σ)_max_ < 0.001
2239 reflections	Δρ_max_ = 0.25 e Å^−^^3^
185 parameters	Δρ_min_ = −0.25 e Å^−^^3^
1 restraint	Extinction correction: none
Primary atom site location: structure-invariant direct methods	

### Special details

**Table:**

Geometry. All e.s.d.'s (except the e.s.d. in the dihedral angle between two l.s. planes) are estimated using the full covariance matrix. The cell e.s.d.'s are taken into account individually in the estimation of e.s.d.'s in distances, angles and torsion angles; correlations between e.s.d.'s in cell parameters are only used when they are defined by crystal symmetry. An approximate (isotropic) treatment of cell e.s.d.'s is used for estimating e.s.d.'s involving l.s. planes.
Refinement. Refinement of *F*^2^ against ALL reflections. The weighted *R*-factor *wR* and goodness of fit *S* are based on *F*^2^, conventional *R*-factors *R* are based on *F*, with *F* set to zero for negative *F*^2^. The threshold expression of *F*^2^ > σ(*F*^2^) is used only for calculating *R*-factors(gt) *etc*. and is not relevant to the choice of reflections for refinement. *R*-factors based on *F*^2^ are statistically about twice as large as those based on *F*, and *R*- factors based on ALL data will be even larger.

### Fractional atomic coordinates and isotropic or equivalent isotropic displacement parameters (Å^2^)

**Table:**

	*x*	*y*	*z*	*U*_iso_*/*U*_eq_	
Cl1	0.76783 (16)	0.49243 (2)	0.80632 (14)	0.1068 (3)	
Cl2	1.33065 (12)	0.06887 (2)	0.76621 (10)	0.0742 (2)	
N1	0.6688 (3)	0.29434 (6)	0.6801 (2)	0.0488 (4)	
N2	0.8017 (3)	0.25847 (6)	0.7096 (2)	0.0523 (5)	
O1	0.2928 (3)	0.33583 (5)	0.5861 (2)	0.0657 (5)	
H1	0.3721	0.3146	0.6045	0.099*	
O2	0.6020 (3)	0.22073 (5)	0.5012 (2)	0.0640 (5)	
C1	0.6271 (3)	0.36918 (6)	0.7166 (3)	0.0445 (5)	
C2	0.4080 (4)	0.37128 (7)	0.6396 (3)	0.0504 (5)	
C3	0.3035 (4)	0.41076 (9)	0.6172 (3)	0.0665 (7)	
H3	0.1576	0.4121	0.5666	0.080*	
C4	0.4116 (5)	0.44766 (9)	0.6685 (4)	0.0729 (7)	
H4	0.3401	0.4740	0.6522	0.087*	
C5	0.6271 (4)	0.44569 (7)	0.7444 (3)	0.0630 (6)	
C6	0.7329 (4)	0.40713 (7)	0.7690 (3)	0.0528 (5)	
H6	0.8779	0.4063	0.8217	0.063*	
C7	0.7485 (4)	0.32942 (7)	0.7410 (3)	0.0468 (5)	
H7	0.8882	0.3295	0.8027	0.056*	
C8	0.7583 (3)	0.22285 (6)	0.6132 (3)	0.0449 (5)	
C9	0.9091 (3)	0.18574 (6)	0.6525 (2)	0.0424 (5)	
C10	0.8262 (4)	0.14516 (7)	0.6112 (3)	0.0481 (5)	
H10	0.6823	0.1423	0.5578	0.058*	
C11	0.9534 (4)	0.10922 (7)	0.6479 (3)	0.0521 (5)	
H11	0.8958	0.0821	0.6218	0.062*	
C12	1.1670 (4)	0.11401 (7)	0.7236 (3)	0.0489 (5)	
C13	1.2546 (4)	0.15368 (7)	0.7646 (3)	0.0509 (5)	
H13	1.3993	0.1563	0.8166	0.061*	
C14	1.1252 (3)	0.18949 (7)	0.7278 (3)	0.0479 (5)	
H14	1.1840	0.2165	0.7538	0.058*	
H2	0.913 (3)	0.2592 (8)	0.795 (3)	0.080*	

### Atomic displacement parameters (Å^2^)

**Table:**

	*U*^11^	*U*^22^	*U*^33^	*U*^12^	*U*^13^	*U*^23^
Cl1	0.1184 (7)	0.0481 (4)	0.1535 (8)	−0.0066 (4)	−0.0009 (6)	0.0049 (4)
Cl2	0.0782 (5)	0.0561 (4)	0.0877 (5)	0.0189 (3)	−0.0070 (4)	−0.0040 (3)
N1	0.0507 (10)	0.0480 (10)	0.0466 (10)	0.0072 (8)	−0.0104 (8)	−0.0010 (8)
N2	0.0576 (12)	0.0464 (10)	0.0512 (11)	0.0081 (9)	−0.0192 (9)	−0.0064 (8)
O1	0.0508 (9)	0.0777 (11)	0.0674 (11)	0.0052 (8)	−0.0123 (8)	−0.0069 (9)
O2	0.0722 (11)	0.0528 (9)	0.0641 (10)	−0.0025 (8)	−0.0340 (9)	−0.0001 (7)
C1	0.0462 (12)	0.0511 (12)	0.0361 (11)	0.0060 (10)	0.0015 (9)	0.0039 (9)
C2	0.0474 (13)	0.0632 (14)	0.0404 (12)	0.0063 (11)	0.0003 (10)	0.0007 (10)
C3	0.0538 (14)	0.0839 (18)	0.0615 (15)	0.0244 (14)	−0.0019 (12)	0.0091 (13)
C4	0.0797 (19)	0.0607 (16)	0.0786 (18)	0.0257 (14)	0.0077 (15)	0.0111 (13)
C5	0.0711 (17)	0.0483 (13)	0.0699 (16)	0.0049 (12)	0.0069 (13)	0.0087 (11)
C6	0.0535 (13)	0.0513 (13)	0.0536 (13)	0.0026 (11)	0.0015 (10)	0.0051 (10)
C7	0.0451 (11)	0.0509 (13)	0.0439 (12)	0.0045 (10)	−0.0065 (9)	0.0019 (9)
C8	0.0491 (12)	0.0444 (11)	0.0406 (11)	−0.0043 (10)	−0.0066 (10)	0.0022 (9)
C9	0.0468 (12)	0.0454 (11)	0.0346 (10)	−0.0013 (9)	−0.0019 (9)	−0.0023 (8)
C10	0.0471 (12)	0.0512 (12)	0.0455 (12)	−0.0030 (10)	−0.0042 (9)	−0.0049 (10)
C11	0.0595 (14)	0.0447 (12)	0.0520 (13)	−0.0051 (11)	0.0036 (11)	−0.0055 (10)
C12	0.0548 (13)	0.0477 (12)	0.0446 (12)	0.0083 (10)	0.0063 (10)	−0.0021 (9)
C13	0.0450 (12)	0.0576 (14)	0.0499 (13)	0.0044 (10)	−0.0023 (10)	−0.0070 (10)
C14	0.0485 (12)	0.0460 (12)	0.0491 (12)	−0.0026 (10)	−0.0014 (10)	−0.0066 (9)

### Geometric parameters (Å, °)

**Table:**

Cl1—C5	1.735 (3)	C4—H4	0.9300
Cl2—C12	1.734 (2)	C5—C6	1.367 (3)
N1—C7	1.270 (3)	C6—H6	0.9300
N1—N2	1.382 (2)	C7—H7	0.9300
N2—C8	1.343 (3)	C8—C9	1.486 (3)
N2—H2	0.898 (10)	C9—C14	1.382 (3)
O1—C2	1.352 (3)	C9—C10	1.390 (3)
O1—H1	0.8200	C10—C11	1.374 (3)
O2—C8	1.222 (2)	C10—H10	0.9300
C1—C6	1.390 (3)	C11—C12	1.373 (3)
C1—C2	1.399 (3)	C11—H11	0.9300
C1—C7	1.443 (3)	C12—C13	1.373 (3)
C2—C3	1.387 (3)	C13—C14	1.377 (3)
C3—C4	1.366 (4)	C13—H13	0.9300
C3—H3	0.9300	C14—H14	0.9300
C4—C5	1.376 (4)		
			
C7—N1—N2	116.23 (17)	N1—C7—H7	119.3
C8—N2—N1	119.42 (17)	C1—C7—H7	119.3
C8—N2—H2	121.1 (16)	O2—C8—N2	122.17 (19)
N1—N2—H2	119.4 (16)	O2—C8—C9	121.69 (18)
C2—O1—H1	109.5	N2—C8—C9	116.13 (17)
C6—C1—C2	118.39 (19)	C14—C9—C10	118.74 (19)
C6—C1—C7	118.83 (19)	C14—C9—C8	123.63 (18)
C2—C1—C7	122.77 (19)	C10—C9—C8	117.63 (18)
O1—C2—C3	118.4 (2)	C11—C10—C9	121.0 (2)
O1—C2—C1	122.06 (19)	C11—C10—H10	119.5
C3—C2—C1	119.5 (2)	C9—C10—H10	119.5
C4—C3—C2	121.0 (2)	C12—C11—C10	118.8 (2)
C4—C3—H3	119.5	C12—C11—H11	120.6
C2—C3—H3	119.5	C10—C11—H11	120.6
C3—C4—C5	119.6 (2)	C11—C12—C13	121.6 (2)
C3—C4—H4	120.2	C11—C12—Cl2	119.07 (17)
C5—C4—H4	120.2	C13—C12—Cl2	119.34 (18)
C6—C5—C4	120.5 (2)	C12—C13—C14	119.1 (2)
C6—C5—Cl1	119.6 (2)	C12—C13—H13	120.4
C4—C5—Cl1	119.96 (19)	C14—C13—H13	120.4
C5—C6—C1	121.0 (2)	C13—C14—C9	120.7 (2)
C5—C6—H6	119.5	C13—C14—H14	119.6
C1—C6—H6	119.5	C9—C14—H14	119.6
N1—C7—C1	121.45 (19)		

### Hydrogen-bond geometry (Å, °)

**Table:**

*D*—H···*A*	*D*—H	H···*A*	*D*···*A*	*D*—H···*A*
N2—H2···O2^i^	0.898 (10)	1.965 (13)	2.826 (2)	160 (2)
O1—H1···N1	0.82	1.93	2.647 (2)	145

Symmetry codes: (i) *x*+1/2, −*y*+1/2, *z*+1/2.
